# Aptamer-Based Diagnostics and Therapeutics

**DOI:** 10.3390/ph12010006

**Published:** 2019-01-02

**Authors:** Sarah Shigdar

**Affiliations:** 1 School of Medicine Deakin University, Geelong, Victoria 3128, Australia; sarah.shigdar@deakin.edu.au; Tel.: +61-03-5227-2846; 2 Centre for Molecular and Medical Research, Deakin University, Geelong, Victoria 3128, Australia

Aptamers were first described almost 30 years ago, with the publication of three separate research papers describing how a randomized library of RNA sequences could be incubated with a target to find a sequence that specifically binds via van der Waals forces, covalent and hydrogen bonding, and not Watson Crick base pairing [1,2,3]. As technology and knowledge have advanced since these initial papers, there have been many developments. These include changes to the selection process, addition of modified bases for better nuclease stability, and the introduction of DNA sequences for aptamers in addition to the first RNA aptamers. Using the simplified high throughput screening of evolving competent binders through iterative cycles of incubation and selection, aptamers have been generated to simple targets such as metal ions, small molecule drugs, and proteins, and more complex targets, such as cells and whole organisms.

Aptamers—also known as chemical antibodies—have been investigated for their utility in many applications encompassing diagnostics and therapeutics. They are very similar to monoclonal antibodies and as such, can be used in any application that antibodies have typically been used in. Given the many unique properties of aptamers, they have been also used in novel assays. In this special issue of Pharmaceuticals, we investigate a number of these novel aspects of aptamers and their future in diagnostics and therapeutics.

It is unsurprising, given the high burden that cancer places, in terms of non-communicable diseases, that the majority of the articles in this special issue review the potential of aptamers for enhanced diagnostics and therapeutic strategies against cancer. Ciancio et al. review how aptamers can be used with a range of current detection and imaging platforms to improve the sensitivity of cancer detection methods. Through the use of nanoparticles, quantum dots, molecular beacons, fluorescent probes, and microfluidic devices, cancer related biomarkers can be detected in a range of complex solutions at an increased level of sensitivity [4]. Following on from this review of detection methods, Gu et al. present a review of the rolling circle amplification (RCA) method and how it can be used to enhance the limit of detection in medical biosensors and biomedical applications against a multitude of targets [5]. Yoon and Rossi focused their review on all aspects of aptamers in molecular imaging of cancer, ranging from live cell imaging in vitro, to in vivo imaging using fluorescence, magnetic nanoparticles, and radiolabelled aptamers [6]. Camorani et al. focused on one specific subtype of cancer, triple negative breast cancer. This type of breast cancer has a poor prognosis and no current targeted therapeutic, though the authors present compelling evidence for the use of aptamers for the treatment of triple negative breast cancer [7].

Continuing with the cancer theme, while providing some applications for aptamers in the detection of infectious diseases, Molefe et al. discuss the use of aptamers for diagnosis and therapeutic applications against cancer, as well as infectious diseases, with particular focus on viral infectious diseases [8].

It is worth noting that several review articles in this special issue discuss radiolabelling aptamers for targeted molecular imaging. The field of molecular imaging and specifically targeting biomarkers for enhanced detection of lesions and tumours is still relatively new and has developed as technology in the clinic has developed. Hassanzadeh et al. present a review on how to radiolabel aptamers, as well as the different types of chelators for attachment, and the various different forms of radioactive isotypes [9]. These aptamers can then be used for both diagnostics and therapeutics. Adding to this, Khalid et al. discuss various aspects of imaging technology as well as comparing antibodies and peptides to aptamers, in order to demonstrate the utility of aptamers in this emerging area [10]. 

Soldevilla et al. have brought together work on numerous nucleic acid therapeutics in their review of aptamer-RNAi delivery. One of the many issues that have prevented RNAi entering the clinic has been the non-specific off-target effects. Aptamers can be used as a targeted agent to ensure RNAi gets to the specific sites it is required to reduce these off-target effects [11]. 

Belleperche and DeRosa present some interesting work on pH-responsive materials, as well as non-canonical nucleic acid base pairing that can shift conformation in response to pH. While this area of aptamer research is still relatively under-researched, there is a lot of untapped potential within this field [12]. 

An interesting application for aptamers presented by Catuogno et al. is epigenetic regulation. Through the targeting of enzymatic regulators responsible for DNA and chromatin modifications, gene transcriptional regulation can be modulated. Aptamers have the potential to be used as innovative tools to investigate the impact of epigenetic mechanisms on gene expression as well as epigenetic modifiers in numerous diseases, including cancer and neurological disorders [13]. 

While aptamers have been developed with a multitude of applications, especially for therapeutic applications, as reviewed in this special issue, Bruno offers a note of caution regarding the unmethylated dinucleotide sequence 2′-deoxycytidine-phosphate-2′-guanine (CpG), which has the potential to stimulate the innate immune system. One of Bruno’s recommendations is to perform toxicity testing if CpG motifs cannot be avoided, as well as offering several strategies of limiting their impact [14]. 

Though it has been nearly 30 years since aptamers were first described, they have been slow to progress into clinical applications. However, within the last few years, we have seen the number of publications with aptamers as a keyword increase, the Aptamer Symposium in Oxford, UK going into its sixth year, the International Society on Aptamers bringing together researchers worldwide, and the Aptamers Journal beginning in 2017. Aptamers are seeing a resurgence and will find their niche in the very near future.

## References

[1] Tuerk C., Gold L. (1990). Systematic evolution of ligands by exponential enrichment: RNA ligands to bacteriophage T4 DNA polymerase. Science.

[2] Ellington A.D., Szostak J.W. (1990). In vitro selection of RNA molecules that bind specific ligands. Nature.

[3] Robertson D.L., Joyce G.F. (1990). Selection in vitro of an RNA enzyme that specifically cleaves single-stranded DNA. Nature.

[4] Ruiz Ciancio D., Vargas R.M., Thiel H.W., Bruno A.M., Giangrande H.P., Mestre B.M. (2018). Aptamers as Diagnostic Tools in Cancer. Pharmaceuticals.

[5] Gu L., Yan W., Liu L., Wang S., Zhang X., Lyu M. (2018). Research Progress on Rolling Circle Amplification (RCA)-Based Biomedical Sensing. Pharmaceuticals.

[6] Yoon S., Rossi J.J. (2018). Targeted Molecular Imaging Using Aptamers in Cancer. Pharmaceuticals.

[7] Camorani S., Fedele M., Zannetti A., Cerchia L. (2018). TNBC Challenge: Oligonucleotide Aptamers for New Imaging and Therapy Modalities. Pharmaceuticals.

[8] Molefe F.P., Masamba P., Oyinloye E.B., Mbatha S.L., Meyer M., Kappo P.A. (2018). Molecular Application of Aptamers in the Diagnosis and Treatment of Cancer and Communicable Diseases. Pharmaceuticals.

[9] Hassanzadeh L., Chen S., Veedu N.R. (2018). Radiolabeling of Nucleic Acid Aptamers for Highly Sensitive Disease-Specific Molecular Imaging. Pharmaceuticals.

[10] Khalid U., Vi C., Henri J., Macdonald J., Eu P., Mandarano G., Shigdar S. (2019). Radiolabelled Aptamers for Theranostic Treatment of Cancer. Pharmaceuticals.

[11] Soldevilla M.M., Meraviglia-Crivelli de Caso D., Menon P.A., Pastor F. (2018). Aptamer-iRNAs as Therapeutics for Cancer Treatment. Pharmaceuticals.

[12] Belleperche M., DeRosa C.M. (2018). pH-Control in Aptamer-Based Diagnostics, Therapeutics, and Analytical Applications. Pharmaceuticals.

[13] Catuogno S., Esposito L.C., Ungaro P., de Franciscis V. (2018). Nucleic Acid Aptamers Targeting Epigenetic Regulators: An Innovative Therapeutic Option. Pharmaceuticals.

[14] Bruno G.J. (2018). Potential Inherent Stimulation of the Innate Immune System by Nucleic Acid Aptamers and Possible Corrective Approaches. Pharmaceuticals.

